# Validation of an Arrhythmogenic Right Ventricular Cardiomyopathy Risk-Prediction Model in a Chinese Cohort

**DOI:** 10.3390/jcm11071973

**Published:** 2022-04-01

**Authors:** Nixiao Zhang, Chuangshi Wang, Alessio Gasperetti, Yanyan Song, Hongxia Niu, Min Gu, Firat Duru, Liang Chen, Shu Zhang, Wei Hua

**Affiliations:** 1 Department of Cardiology, Cardiovascular Center, Beijing Friendship Hospital, Capital Medical University, Beijing 100050, China; zhangnx_doc@163.com; 2 Cardiac Arrhythmia Center, State Key Laboratory of Cardiovascular Disease, Fuwai Hospital, National Center for Cardiovascular Disease, Chinese Academy of Medical Sciences and Peking Union Medical College, Beijing 100037, China; drniu@126.com (H.N.); gumin1012@163.com (M.G.); zhangshufw@163.com (S.Z.); 3 Medical Research and Biometrics Center, Fuwai Hospital, National Center for Cardiovascular Disease, Chinese Academy of Medical Sciences and Peking Union Medical College, Beijing 102300, China; wangchuangshi@mrbc-nccd.com; 4 Department of Cardiology, University Heart Center Zurich, University Hospital Zurich, 8091 Zurich, Switzerland; alessio.gasperetti93@gmail.com (A.G.); firat.duru@usz.ch (F.D.); 5 Department of CMR, State Key Laboratory of Cardiovascular Disease, Fuwai Hospital, National Center for Cardiovascular Diseases, Chinese Academy of Medical Sciences and Peking Union Medical College, Beijing 100037, China; songyy1011@163.com; 6 Department of Cardiac Surgery, State Key Laboratory of Cardiovascular Disease, Fuwai Hospital, National Center for Cardiovascular Diseases, Chinese Academy of Medical Sciences and Peking Union Medical College, Beijing 100037, China

**Keywords:** arrhythmogenic cardiomyopathy, primary prevention, secondary prevention, implantable cardioverter-defibrillator, ventricular arrhythmias, recalibration

## Abstract

Background: The novel arrhythmogenic right ventricular cardiomyopathy (ARVC)-associated ventricular arrhythmias (VAs) risk-prediction model endorsed by Cadrin-Tourigny et al. was recently developed to estimate visual VA risk and was identified to be more effective for predicting ventricular events than the International Task Force Consensus (ITFC) criteria, and the Heart Rhythm Society (HRS) criteria. Data regarding its application in Asians are lacking. Objectives: We aimed to perform an external validation of this algorithm in the Chinese ARVC population. Methods: The study enrolled 88 ARVC patients who received implantable cardioverter-defibrillator (ICD) from January 2005 to January 2020. The primary endpoint was appropriate ICD therapies. The novel prediction model was used to calculate a priori predicted VA risk that was compared with the observed rates. Results: During a median follow-up of 3.9 years, 57 (64.8%) patients received the ICD therapy. Patients with implanted ICDs for primary prevention had non-significantly lower rates of ICD therapy than secondary prevention (5-year event rate: 0.46 (0.13–0.66) and 0.80 (0.64–0.89); log-rank *p* = 0.098). The validation study revealed the C-statistic of 0.833 (95% confidence interval (CI) 0.615–1.000), and the predicted and the observed patterns were similar in primary prevention patients (mean predicted–observed risk: −0.07 (95% CI −0.21, 0.09)). However, in secondary prevention patients, the C-statistic was 0.640 (95% CI 0.510–0.770) and the predicted risk was significantly underestimated (mean predicted–observed risk: −0.32 (95% CI −0.39, −0.24)). The recalibration analysis showed that the performance of the prediction model in secondary prevention patients was improved, with the mean predicted–observed risk of −0.04 (95% CI −0.10, 0.03). Conclusions: The novel risk-prediction model had a good fitness to predict arrhythmic risk in Asian ARVC patients for primary prevention, and for secondary prevention patients after recalibration of the baseline risk.

## 1. Introduction

Arrhythmogenic right ventricular cardiomyopathy (ARVC) is an inherited cardiomyopathy that is characterized by fibro-fatty replacement of the myocardium and related to a higher risk of ventricular arrhythmias (VAs) and sudden cardiac death (SCD) [1,2]. In some individuals, SCD is the first and the only manifestation of ARVC, which accounts for about 16% of the incidence in ARVC [1]. Once ARVC is diagnosed, the stratification of the SCD risk is of great significance and the prevention of SCD with implantable cardioverter-defibrillations (ICDs) is the cornerstone of ARVC management [3]. For ARVC patients who have a history of an aborted SCD or who have recorded sustained ventricular tachyarrhythmias (VTs) with hemodynamic instability, an ICD is strongly recommended [4].

Recently, Cadrin-Tourigny et al. [5] established a new 5-year risk-prediction model for VAs consisting of sex, age, history of recent cardiac syncope, history of non-sustained VT, 24 h premature ventricular contraction count (PVC), the sum of anterior and inferior leads with T-wave inversion (TWI), and right ventricular ejection fraction (RVEF) in the transatlantic ARVC cohort, and proved it superior to the current consensus-based ICD implantation algorithm and International Task Force Consensus (ITFC) model. Afterwards, this novel model was validated by different European ARVC cohorts [6,7,8]. Recently, Cadrin-Tourigny et al. reassessed the predictors of life-threatening VAs in ARVC patients regardless of prior VAs and four (younger age, male sex, PVC count, and the number of leads with TWI) had significant associations [9]. Several pathogenic variants’ frequencies differ across seven ethnic groups (Non-Finnish European, Finnish, East Asian, South Asian, Latino, African, and Ashkenazi Jewish) [10]. It is possible that the racial diversity in ARVC-associated VA risk could influence the predictive power of this novel model in different races. However, whether this novel model can be applied to Asian patients with ARVC is still unknown, and its performance in patients for secondary prevention is not well examined.

In the present study, we aimed to validate the efficacy of the ARVC risk model in a Chinese patient cohort and explore the clinical application to secondary prevention patients.

## 2. Methods

### 2.1. Patient Cohort

All of the 119 patients with a definite ARVC diagnosis who received their first ICD implantation at the department of Cardiac Arrhythmia Center, Fuwai Hospital (Beijing, China) were consecutively enrolled in the study from January 2005 to January 2020. Four patients were excluded due to known associated obstructive coronary artery disease, including a history of myocardial infarction. Eighty-eight patients also undergoing the Cardiac Magnetic Resonance (CMR) examination before the ICD implantation were finally included for further analysis (Supplemental Figure S1). All the patients provided written informed consent, and the study was approved by the local ethics committee and complied with the Declaration of Helsinki.

### 2.2. Definitions

ARVC was diagnosed according to the 2010 Revised Task Force Criteria when 2 major, or 1 major plus 2 minor, or 4 minor criteria from different categories were met [11].

### 2.3. Baseline Evaluation

Before the ICD implantation, all enrolled patients routinely underwent a 12-lead electrocardiogram (ECG), 24-h Holter ECG monitoring, routine cardiac ultrasound, and CMR.

All CMR images were obtained with a 1.5-T MAGNETOM Avanto (Siemens Healthcare, Erlangen, Germany), 3.0-T MR750 (GE Healthcare, Chicago, IL, USA), and 3.0 T Ingenia (Philips, Amsterdam, The Netherlands) clinical scanner, using electrocardiographic and respiratory gating. Transversal and sagittal dark blood imaging were performed using a half-Fourier acquisition single-shot turbo spin-echo sequence. Typical parameters: field of view (FOV), 340 × 280 mm^2^; matrix, 256 × 113; slice thickness, 6–8 mm; repetition time (TR), two or three heartbeats; echo time (TE), 42 ms. LV cine images were acquired in 3 long-axis views including LV 2-chamber, 4-chamber, and LV outflow tract (LVOT), and continuously used a stack of 8–10 short-axis slices covering the entire LV by applying segmented balanced steady-state free precession (b-SSFP) sequence. The typical imaging parameters included: TR = 2.8–3.0 ms, TE = 1.1–1.5 ms, flip angle = 60–70°, temporal resolution = 30–55 ms, FOV = 360 × 315 mm^2^, matrix = 192 × 162, slice thickness = 8 mm, slice gap = 2 mm. The software used for MRI cine images post-processing was Argus (Siemens Company, Munich, Germany). Epicardial and endocardial borders of LV/RV myocardium were manually traced in all phases on short-axis cine images in order to calculate LVEF/RVEF.

### 2.4. ICD Implantation and Interrogation

An ICD was implanted intravenously. The passive atrial lead was located at the right auricle and the active fixation ventricular lead was screwed into the septum near the right ventricular outflow tract. Defibrillation thresholds were not routinely tested during the operation. Decisions regarding ICD types, implantation, and programming were made at the discretion of the charge cardiologists. Programmed anti-tachycardia pacing (ATP) or discharge or both was activated as soon as the operation was completed successfully.

Available stored intracardiac electrograms were analyzed to classify arrhythmias in order to precipitate defibrillation discharges, according to a prior study [12]. VF or ventricular flutter was defined as regular or irregular tachycardia concerning QRS morphology, amplitude, and sequence, with a mean cycle length of 240 ms or less. VT was defined as regular (monomorphic) or irregular (polymorphic) tachycardia with a mean cycle length of more than 240 ms.

### 2.5. Arrhythmia Event Evaluation and Follow-Up

Through inquiry, the family history of SCD, recent cardiac syncope, and all the prior arrhythmic events including NSVT, sustained VT, and ventricular fibrillation/flutter (VF) at baseline were assessed. The 24-h Holter monitoring was extracted to record the 24-h PVC count. 

The study was initiated on the day of the ICD implantation. Patients routinely underwent follow-up for ICDs programming at 3 months and 6 months after hospital discharge, and then every 12 months thereafter, or immediately after the feelings of cardiac symptoms (such as palpitations, dizziness, syncope, etc.), and ICD defibrillations. Telephone follow-up was available for patients who could not come to the clinic. The follow up time was capped on 30 June 2020.

### 2.6. Study Outcomes

The primary outcome of the study was the first ICD-appropriate therapy composed of anti-tachyarrhythmia pacing (ATP) and automatic defibrillation shocks due to sustained VTs and VFs by stored intracardiac electrograms. For patients who had defibrillations with stored electrographic data eliminated because of the data-cleaning process before the current interrogation, discharged patients were judged to be appropriate on the basis of clinical findings (i.e., presyncope or syncope immediately before the discharge and the absence of the findings immediately afterward) that strongly suggested the presence of VAs. The secondary endpoint was cardiac death, including heart transplantation.

The predicted VA rate was calculated using the Cadrin-Tourigny et al. prediction model and compared to the observed ICD therapy rate during follow-up. The 5-year sustained VA risk for individual patients was calculated: P (VA at 5 years) = 1 − 0.801^exp (LP)^ where LP = 0.488 × sex − 0.022 × age + 0.657 × history of recent cardiac syncope + 0.811 × history of NSVT + 0.170 × ln(24 h PVC count) + 0.113 × sum of anterior and inferior leads with T-wave inversion − 0.025 × RVEF [5]. Yearly risk was calculated with the values of the baseline indicated parameters. 

### 2.7. Statistical Analysis

All statistical analyses were performed using SPSS version 25 (IBM, Armonk, NY, USA) and R Project version 3.6. Continuous variables were presented as mean ± standard deviation or median (interquartile range, IQR) and the comparison between different groups was performed using the *t*-test or the Mann–Whitney U test, as appropriate. Categorical variables were described as count (percentage). The χ^2^-test was used to compare categorical variables between different groups. Missing values of the 24-h Holter PVC count were imputed by the median. Kaplan–Meier curves were used to depict rates of ICD therapy, and the cardiac death and differences between patients for primary prevention and secondary prevention were compared using the log-rank test. The performance of the novel risk model was evaluated by the C-statistic, which is considered as an indicator for discriminating high from low risk of sustained VAs in ARVC. Moreover, the differences of predicted and observed rates of VAs were computed to evaluate its calibration. 

A recalibration of the ARVC risk model was performed for application in the risk prediction of the secondary prevention subpopulation by updating the baseline survival probability. The baseline survival probability was re-calibrated based on the secondary prevention population in our study by comparing the predicted survival with the observed survival on the log hazard scale to correct “calibration-in-the-large” [13,14]. History of sustained VT/VF was used to classify patients into primary or secondary prevention populations. All tests were two-tailed, and *p* < 0.05 was deemed statistically significant.

## 3. Results

### 3.1. Baseline Characteristics

A total of 88 ARVC patients (mean age 42.4 ± 14.1 years, 71.6% male) who underwent the ICD implantation were available for further analyses. Most of the baseline features between the included and excluded patients had no significance, except the administration of renin-angiotensin system inhibitors (Supplemental Table S1). Table 1 lists the baseline characteristics of all included patients and the comparison between those with and without the appropriate ICD therapy during follow-up. Recent cardiac syncope within 6 months on admission was seen in 29 (33.0%) patients. A history of NSVT was found in 44 (50.0%) patients. The median sum of anterior and inferior leads with TWI was 3 (IQR, 2–5). The median PVC count during the 24-h ambulatory monitoring was 1399 (IQR 593–2742). Furthermore, the mean RVEF and LVEF were 27.6 ± 14.4%, and 49.2 ± 12.4%, respectively. Genetic testing was performed in 16 (18.2%) patients, and nine were positive for the pathogenic mutations, of which *DSG2* and *PKP2* were the most common pathogenic genes. Of all 88 patients, about 81% (*n* = 71) had an implanted ICD for secondary prevention, and 19% for primary prevention.

### 3.2. Follow-Up Analysis

**Figure 1:**
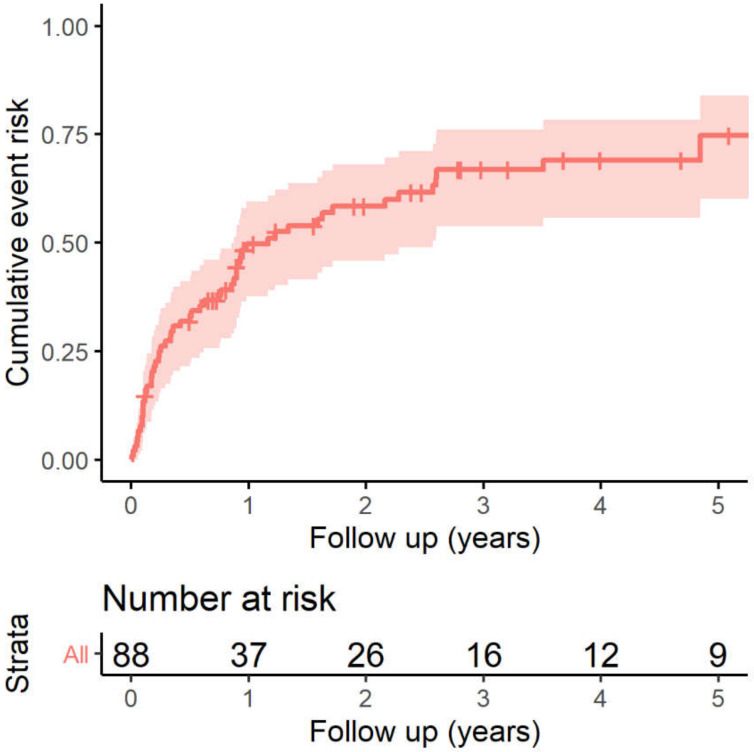
Kaplan–Meier analysis of the appropriate ICD therapy for total population. ICD, implantable cardioverter-defibrillator.

During a median follow-up of 3.9 (IQR 1.6–6.9) years, the first appropriate ICD therapy was recorded in 57 (64.8%) patients. Among 17 patients who had an ICD implanted for primary prevention, 8 (47.1%) patients came to the primary endpoint; and in those for the secondary prevention, 49 (69.0%) received appropriate ICD therapy. The overall event rate of the ICD therapy was 0.50 (95% confidence interval (CI) 0.38–0.60) at 1 year, 0.58 (0.46–0.68) at 2 years and 0.75 (0.60–0.84) at 5 years, respectively. The cumulative event risk of the population is shown in Figure 1. Thus, we next studied the clinical characteristics of patients with or without ICD-appropriate therapy during follow-up among the secondary prevention patients. The baseline characteristics by ICD therapy for the subgroups are shown in Table 2.

### 3.3. Validation of the 5-Year Risk-VA Model

**Figure 2:**
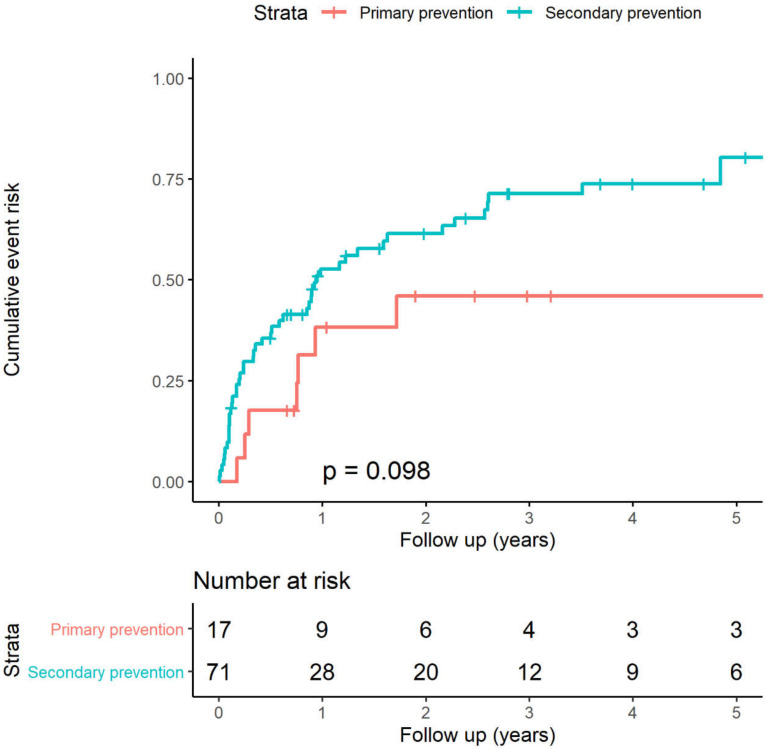
Kaplan–Meier analysis of the appropriate ICD therapy stratified by ICD indications. ICD, implantable cardioverter-defibrillator.

**Figure 3:**
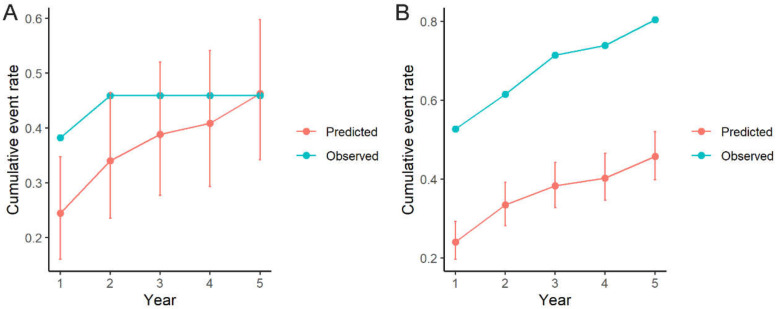
Comparison between model-predicted and observed event rates in patients implanted with ICDs for (**A**) primary prevention of SCD; (**B**) secondary prevention of SCD. ICD, implantable cardioverter-defibrillator. SCD, sudden cardiac death.

The C-statistic for the ARVC-Risk-VA model was 0.681 (95% CI 0.567–0.796) in this Chinese cohort. The comparison between predicted and observed event rates is depicted in Supplemental Figure S2. A significant difference in ICD therapy rates between the predicted and the observed patterns was found during follow-up (mean predicted–observed rate: −0.27 (95% CI −0.33, −0.22)) in the whole population. Given that our cohort is remarkedly distinguished from that used for model development in western institutions, the patients were further categorized into two subgroups according to the medical history of the sustained VT/VF and then re-analyzed. Patients who received ICD implantation for the primary prevention had the lower LVEF (38.8 ± 12.9 versus 51.6 ± 11.0, *p* < 0.001), and the higher percentage of NSVT (76.5% versus 43.7%, *p* = 0.029). Survival curves for patients stratified by primary and secondary prevention are reported in Figure 2, and a notable difference in the ICD therapy was observed between the two groups (5-year event rate: 0.46 (0.13–0.66) and 0.80 (0.64–0.89) for primary prevention and secondary prevention, respectively), despite the statistical insignificance due to small sample size in both groups (log-rank *p* = 0.098). The C-statistic of this prediction model was 0.833 (95% CI 0.615–1.000) and 0.640 (95% CI 0.510–0.770) for the primary and secondary prevention population, respectively. Figure 3 reports the difference between predicted and observed event rates stratified by primary and secondary prevention patients: there was no significant difference between the predicted and the observed ICD therapies in the non-sustained VT/VF group (i.e., the primary prevention population) (mean difference predicted–observed rate: −0.07 (95% CI −0.21, 0.09); Figure 3A). The prediction model underestimated the observed ICD therapies (mean difference predicted–observed rate: −0.32 (95% CI −0.39, −0.24); Figure 3B) in the sustained VT/VF group (i.e., the secondary prevention population).

### 3.4. Recalibration and Validation of Risk Model in Secondary Prevention Patients

As secondary prevention patients are expected to have higher risks than primary prevention patients and the systematically underestimated risks were shown for this subpopulation in our analysis, we further conducted the recalibration by updating the baseline survival probability to improve the prediction for this specific group of patients. 

For an individual patient, the risk of the appropriate ICD therapy for 5 years was calculated using the following modified equation:

P (VA at 5 years) = 1 − 0.801 0.512^exp (LP)^

where LP = 0.488 × sex − 0.022 × age + 0.657 × history of recent cardiac syncope + 0.811 × history of NSVT + 0.170 × ln(24 h PVC count) + 0.113 × sum of anterior and inferior leads with TWI − 0.025 × RVEF.

Table 3 provides the probability of survival (S_0_(t)) at 1, 2, 3, and 4 years to facilitate the calculation of risk for shorter time durations before and after recalibration.

**Figure 4:**
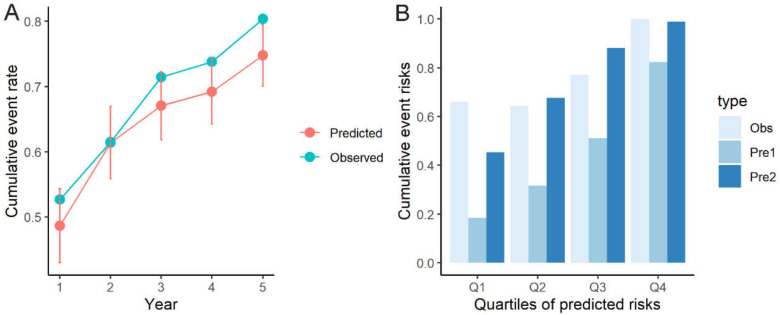
Comparison between model-predicted and observed event rates after recalibration (**A**); and comparisons in cumulative observed, predicted before and after recalibration events rates in patients implanted with ICDs for secondary prevention (**B**). ICD, implantable cardioverter-defibrillator.

After recalibration, there were no significant differences between the predicted and the observed ICD therapy rates during the follow-up: −0.04 ((95% CI −0.10, 0.03); Figure 4A). The calibration plot by quartiles of predicted risk also showed no substantial difference between recalibrated prediction rates (dark blue bars) and observed rates and great improvement compared with those before recalibration (light blue bars) (Figure 4B).

### 3.5. Sensitivity Analysis

After excluding patients with missing values of 24 h PVC count, significant differences were also found in ICD therapy between the predicted and the observed rates in all patients (mean difference predicted–observed rate: −0.27, 95% CI −0.33, −0.21) and in the secondary prevention population (mean difference predicted–observed rate: −0.31, 95% CI −0.37, −0.25). After recalibration using the same method as in the main analysis, no significant difference was observed between predicted and observed rates (−0.03, 95% CI −0.10, 0.04).

### 3.6. Prognosis between Primary and Secondary Prevention

During the whole follow-up time (4.8 ± 3.3 years), three (17.6%) had cardiac death among the primary prevention patients; in the secondary prevention patients, six (8.4%) had cardiac death. Kaplan–Meier analysis revealed no significant difference between primary and secondary prevention in terms of cardiac death rates (log-rank *p* = 0.221; Supplemental Figure S3). In patients who had ICD therapy, 15.8% (9/57) patients reached the secondary endpoint; and in those without ICD therapy, no one suffered cardiac death. A significant difference was found in the cardiac death rates between patients with and without ICD therapy (log-rank *p* = 0.047). However, there were no significant differences in cardiac death without and with the ICD therapy when further analyzing the primary prevention (0 vs. 37.5%, *p* = 0.082) and secondary prevention populations (0 vs. 12.2%, *p* = 0.098).

## 4. Discussion

In the past 20 years, multiple studies have identified factors associated with VA among patients with ARVC, including genetics [15], clinical variables [5,16], as well as biomarkers [17,18,19]. Our present investigation is the first study to validate the ARVC 5-year Risk-VAs calculator endorsed by Cadrin-Tourigny et al. [5] in 2019 in an independent cohort of the Chinese ARVC population. The main findings of the current study included the following: the ARVC risk model worked well for primary prevention patients, and the goodness-of-fit was greatly improved after recalibration and applied successfully for secondary prevention patients in the Chinese ARVC population.

The ARVC 5-year Risk-VAs calculator proposed by a collaborative team included seven predictors of sex, age, history of recent cardiac syncope, history of NSVT, 24 h PVC count, the sum of anterior and inferior leads with TWI, and RVEF [5]. This multi-center study included 528 patients with definite ARVC and no history of sustained VA or sudden cardiac death from North America and European countries. During a median follow-up of 4.83 years, 146 (27.7%) patients experienced sustained VAs. The optimism-corrected C-statistic was 0.77 (95% CI 0.73–0.81) and the calibration slope of internal validation was 0.93 (95% CI 0.92–0.95). This novel prediction model was able to generate individualized estimates of the risk of VA based on readily available clinical variables. Furthermore, this model was validated in multiple independent cohorts.

A subsequent validation study from Italy contained 101 patients who were diagnosed with ARVC with at least a ‘borderline’ level [7]. This study contained both primary and secondary prevention patients. On admission, history and documented sustained VAs were found in 15 (14.9%) patients, and on discharge, 68 (67.3%) patients underwent ICD implant. During a median follow-up of 5.4 (2.6–8.4) years, 43 (42.6%) patients had sustained VA events. It was reported that the Cadrin-Tourigny et al. predictive model effectively described the overall cohort risk and, in particular, classical RV-dominated ARVC, but seemed to underestimate it in non-classical subtypes. Physical exercise can greatly increase arrhythmic risk in ARVC patients [20,21], and thus, the validation of the Cadrin-Tourigny et al. algorithm in the athlete cohort is interesting. In a validation study including 25 athletes with a definitive ARVC diagnosis, five patients (20%) had documented sustained Vas [6]. After disease diagnosis, 10 (40%) ICD implantations were performed. During a median follow-up of 5.3 (3.2–6.6) years, 10 athletes (40%) had a sustained VA event. There was no significant difference between algorithm-predicted and observed rates, suggesting a good prediction performance of the ARVC risk model among athletes.

In addition, a multinational collaboration from 15 centers involving 864 definite ARVC patients re-evaluated the clinical value of the predictors in the prediction model and reported the association of younger age, male sex, PVC count, and the number of anterior and inferior leads with TWI with life-threatening Vas [9]. However, in this study, 38.8% of patients had a history of sustained VAs at the time of diagnosis and 43.4% of patients experienced any sustained VA event during a median follow-up of 5.75 years. Thus, it is reasonable to separately validate the prediction model in patients without prior VAs and with prior VAs.

Given that the ARVC 5-year Risk-VAs calculator was based on transatlantic cohorts, its application and validation in the Asian population remain lacking. The ARVC cohorts from China contain a higher proportion of advanced stages [22,23,24], and most cases received ICD implantation for secondary prevention, which is different from the situation in western countries. Furthermore, a large proportion of cases with a history of sustained VAs are still willing to receive catheter ablation instead of ICD implantation in China, even though most ARVC patients can benefit from the implantation of ICDs [4,25]. Our study showed a higher proportion of secondary prevention patients (80.7% vs. 38.8%) and higher event rates (total: 64.8% vs. 10.8%; with prior VA: 69.0% vs. 11.9%; without prior VA: 47.1% vs. 10.0%) than the western cohort in ARVC [9,26]. Thus, in the present study, we validated the predictability of the ARVC 5-year Risk-VAs prediction model in the Chinese ARVC cohort, and applied the model to primary and secondary prevention subgroups separately. Our data confirmed that the novel prediction model well predicted the VAs risk of ARVC patients without histories of VAs in the Chinese population, whereas the predicted efficacy of this model was underestimated when applied to ARVC patients who suffered VAs before admission. We further performed a recalibration of the model to adjust the risk basis for the secondary prevention patients, and the validation results indicated that the recalibrated model was well fitted to the ARVC patients with a history of VAs, using the eight pre-specified clinical predictors. 

In our study, the application effect of the prediction model in the total population did not reach the expectation, and we finally divided the population into primary and secondary prevention groups for research. This was because we took into account that the characteristics of these two populations were different in the Chinese ARVC cohort as compared to the western cohort [9,26] and that the predictive statistics of the model based on these two populations might change based on the ratio of secondary prevention patients and primary prevention patients. In addition, there were some differences in the clinical evaluation between the two groups [4]. Therefore, based on clinical practices, our cohort was divided into primary prevention patients and secondary prevention patients for the validation of applicability of the novel model, and the results performed well, although re-calibration was required in secondary prevention patients.

### Study Limitation

This was a single-center, retrospective study based on a tertiary referral center, so selection bias was inevitable. The appropriate ICD therapy was not equivalent to the VAs, Nevertheless, the intracardiac recordings were relatively accurate when a retrospective study was performed. The study was also limited by the relatively small cohort, especially the subgroup for primary prevention. However, the prediction model performed well even in this small-size subgroup.

## 5. Conclusions

The novel Caudrin-Tourigny et al. ARVC-VA-Risk model showed a good fitness for the prediction of arrhythmic risk among our external ARVC patients without prior VAs. Furthermore, this model was successfully extended to the secondary prevention patients after the recalibration of the baseline risk. Therefore, the application of the novel model and its recalculated form to primary and secondary prevention patients is promising in guiding ICD placement among Asian patients with ARVC.

## Figures and Tables

**Table 1:** Baseline clinical characteristics between patients with and without appropriate ICD therapy.

	All Patients (*n* = 88)	Appropriate ICD Therapy		*p* Value
		No (*n* = 31)	Yes (*n* = 57)	
**Age at implantation, y**	**42.4 ± 14.1**	44.5 ± 14.0	41.3 ± 14.2	0.302
**Male, *n* (%)**	63 (71.6)	19 (61.3)	44 (77.2)	0.114
**BMI, kg/m^2^**	23.9 ± 3.3	24.0 ± 3.2	23.9 ± 3.4	0.896
**Family history of SCD, *n* (%)**	9 (10.2)	4 (12.9)	5 (8.8)	0.808
**Recent cardiac syncope, *n* (%)**	29 (33.0)	13 (41.9)	16 (28.1)	0.186
**Medical history, *n* (%)**				
AF	10 (11.4)	2 (6.5)	8 (14.0)	0.472
Hypertension	11 (12.5)	3 (9.7)	8 (14.0)	0.555
DM	1 (1.1)	0	1 (1.8)	>0.999
Sustained VT/VF	71 (80.7)	22 (71.0)	49 (86.0)	0.089
NSVT	44 (50.0)	13 (41.9)	31 (54.4)	0.265
**ECG features, *n* (%)**				
RBBB	18 (20.5)	5 (16.1)	13 (22.8)	0.458
Extensive TWI	55 (62.5)	16 (51.6)	39 (68.4)	0.120
Sum of anterior and inferior leads with TWI	3 (2–5)	3 (1–5)	4 (2–5)	0.238
24 h PVCs count	1399 (593–2742)	1399 (247–2160)	1399 (670–4311)	0.100
**CMR features**				
LVEF, %	49.2 ± 12.4	51.1 ± 13.1	48.1 ± 12.0	0.281
RVEF, %	27.6 ± 14.4	32.6 ± 15.3	24.8 ± 13.2	0.014
**Drug administration, *n* (%)**				
β-receptor blockers	46 (52.3)	18 (58.1)	28 (49.1)	0.422
Other AADs	72 (81.8)	26 (83.9)	46 (80.7)	0.713
ACEI/ARB/ARNI	43 (48.9)	16 (51.6)	27 (47.4)	0.704
**Single-chamber ICD, *n* (%)**	70 (79.5)	24 (77.4)	46 (80.7)	0.715

Categorical variables are presented as *n* (%). Continuous variables are presented as mean ± standard deviation or median (interquartile range). BMI, body mass index; SCD, sudden cardiac death; AF, atrial fibrillation; DM, diabetes mellitus; VT, ventricular tachyarrhythmia; VF, ventricular fibrillation; NSVT, non-sustained ventricular tachyarrhythmia; ECG, electrocardiogram; RBBB, right bundle branch block; TWI, T-wave inversion; PVCs, premature ventricular complexes; CMR, cardiac magnetic resonance; LVEF, left ventricular ejection fraction; RVEF, right ventricular ejection fraction; AADs, anti-arrhythmia drugs; ACEI/ARB/ARNI, angiotensin-converting enzyme inhibitor/angiotensin receptor blocker/angiotensin receptor neprilysin inhibitor; ICD, implantable cardioverter-defibrillator.

**Table 2:** Comparisons between secondary prevention patients with and without ICD therapy.

	Secondary Prevention Patients (*n* = 71)	Appropriate ICD Therapy		*p* Value
		No (*n* = 22)	Yes (*n* = 49)	
**Age at implantation, y**	**42 ± 13**	44.8 ± 11.3	41.4 ± 13.8	0.314
**Male, *n* (%)**	53 (74.6)	13 (59.1)	40 (81.6)	0.074
**BMI, kg/m^2^**	23.8 ± 3.1	23.7 ± 3.0	23.8 ± 3.2	0.872
**Family history of SCD, *n* (%)**	6 (8.5)	2 (9.1)	4 (8.2)	>0.999
**Recent cardiac syncope, *n* (%)**	26 (36.6)	11 (50.0)	15 (30.6)	0.182
**Medical history, *n* (%)**				
AF	6 (8.5)	0	6 (12.2)	0.167
Hypertension	9 (12.7)	2 (9.1)	7 (14.3)	0.711
DM	1 (1.4)	0	1 (2.0)	>0.999
NSVT	31 (43.7)	7 (31.8)	24 (49.0)	0.205
**ECG features, *n* (%)**				
RBBB	14 (19.7)	4 (18.2)	10 (20.4)	>0.999
Extensive TWI	45 (63.4)	12 (54.5)	33 (67.3)	0.425
Sum of anterior and inferior leads with TWI	4 (2–5)	3 (2–5)	4 (2–5)	0.576
24 h PVCs count	1399 (577–2686)	1399 (603–2415)	1399 (572–3322)	0.562
**CMR features**				
LVEF, %	51.6 ± 11.0	55.2 ± 10.7	50.0 ± 10.9	0.068
RVEF, %	27.7 ± 13.5	31.4 ± 13.0	26.0 ± 13.5	0.119
**Drug administration, *n* (%)**				
β-receptor blockers	34 (47.9)	10 (45.5)	24 (49.0)	0.803
Other AADs	63 (88.7)	21 (95.5)	42 (85.7)	0.420
ACEI/ARB/ARNI	31 (43.7)	9 (40.9)	22 (44.9)	0.801
**Single-chamber ICD, *n* (%)**	58 (81.7)	18 (81.8)	40 (81.6)	>0.999

Categorical variables are presented as *n* (%). Continuous variables are presented as mean ± standard deviation or median (interquartile range). BMI, body mass index; SCD, sudden cardiac death; AF, atrial fibrillation; DM, diabetes mellitus; VT, ventricular tachyarrhythmia; VF, ventricular fibrillation; NSVT, non-sustained ventricular tachyarrhythmia; ECG, electrocardiogram; RBBB, right bundle branch block; TWI, T-wave inversion; PVCs, premature ventricular complexes; CMR, cardiac magnetic resonance; LVEF, left ventricular ejection fraction; RVEF, right ventricular ejection fraction; AADs, anti-arrhythmia drugs; ACEI/ARB/ARNI, angiotensin-converting enzyme inhibitor/angiotensin receptor blocker/angiotensin receptor neprilysin inhibitor; ICD, implantable cardioverter-defibrillator.

**Table 3:** Predicted probability of survival for different follow-up durations.

Time	Probability of Survival (S_0_(t)) after Recalibration	Probability of Survival (S_0_(t)) Reported by Cadrin-Tourigny
1-year	0.780	0.921
2-year	0.671	0.876
3-year	0.610	0.849
4-year	0.584	0.837
5-year	0.512	0.801

Taking 5-year risk as an example, P (VA at 5 years) = 1 − 0.801 0.512^exp (LP)^ where LP = 0.488 × sex − 0.022 × age + 0.657 × history of recent cardiac syncope + 0.811 × history of NSVT + 0.170 × ln(24 h PVC count) + 0.113 × sum of anterior and inferior leads with T-wave inversion − 0.025 × RVEF.

## Data Availability

Data supporting this study are available upon reasonable request to the corresponding author.

## Supplementary Materials

The following supporting information can be downloaded at: https://www.mdpi.com/article/10.3390/jcm11071973/s1, Figure S1: The flowchart of study design. ARVC, arrhythmogenic right ventricular cardiomyopathy; ICD, implantable cardiovert-er-defibrillator; Figure S2: Comparison between model-predicted and observed event rates in total patients; Figure S3: Comparison of the cardiac death stratified by primary prevention and secondary prevention; Table S1: Comparisons of baseline features between included and excluded patients.
