# Supplementary material for: Validation of an Arrhythmogenic Right Ventricular Cardiomyopathy Risk-Prediction Model in a Chinese Cohort
Source: J Clin Med. 2022 Apr 1;11(7):1973. doi: 10.3390/jcm11071973 (PMC8999693; doi:10.3390/jcm11071973)
Supplement: Supplementary file 1 [file jcm-11-01973-s001.zip › jcm-1646295-supplementary.pdf]

Supplementary Materials

Table S1. Comparisons of baseline features between included and excluded patients.

	All patients (n=119)	Patients Excluded (n=31)	Included (n=88)	P value
**Age at implantation, y**	**42.4±14.2**	42.6±14.6	42.4±14.1	0.960
**Male, n (%)**	86 (72.3)	23 (74.2)	63 (71.6)	0.821
**BMI, kg/m^2**	24.0±3.3	24.3±3.3	23.9±3.3	0.557
**Family history of SCD, n (%)**	12 (10.1)	3 (9.7)	9 (10.2)	>0.999
**Recent cardiac syncope, n (%)**	42 (35.3)	13 (41.9)	29 (33.0)	0.389
**Medical history, n (%)**				
AF	13 (10.9)	3 (9.7)	10 (11.4)	>0.999
Hypertension	16 (13.4)	5 (16.1)	11 (12.5)	0.760
DM	2 (1.7)	1 (3.2)	1 (1.1)	0.455
Sustained VT/ VF	98 (82.4)	27 (87.1)	71 (80.7)	0.586
NSVT	58 (48.7)	14 (45.2)	44 (50.0)	0.681
**ECG features, n (%)**				
RBBB	29 (24.4)	11 (35.5)	18 (20.5)	0.143
Extensive TWI	78 (65.5)	23 (74.2)	55 (62.5)	0.278
Sum of anterior and inferior leads with TWI	4 (2-5)	4 (2-8)	3 (2-5)	0.105
24h PVCs count	1399 (461-2705)	837 (107-2591)	1399 (593-2742)	0.173
**CMR features**				
LVEF, %	-	-	49.2±12.4	-
RVEF, %	-	-	27.6±14.4	-
**Drug administration, n (%)**				
β-receptor blockers	63 (52.9)	17 (54.8)	46 (52.3)	0.837
Other AADs	101 (84.9)	29 (93.5)	72 (81.8)	0.151
ACEI/ARB/ARNI	51 (42.9)	8 (25.8)	43 (48.9)	0.034
**Single-chamber ICD, n (%)**	93 (78.2)	23 (74.2)	70 (79.5)	0.614

Categorical variables are presented as n (%). Continuous variables were presented as mean ± standard deviation or median (interquartile range). BMI, body mass index; SCD, sudden cardiac death; AF, atrial fibrillation; DM, diabetes mellitus; VT, ventricular tachyarrhythmia; VF, ventricular fibrillation; NSVT, non-sustained ventricular tachyarrhythmia; ECG, electrocardiogram; RBBB, right bundle branch block; TWI, T wave inversion; PVCs, premature ventricular complexes; CMR, cardiac magnetic resonance; LVEF, left ventricular ejection fraction; RVEF, right ventricular ejection fraction; AADs, anti-arrhythmia drugs; ACEI/ARB/ARNI, angiotensin-converting enzyme inhibitor/ angiotensin receptor blocker/ angiotensin receptor neprilysin inhibitor; ICD, implantable cardioverter-defibrillator.

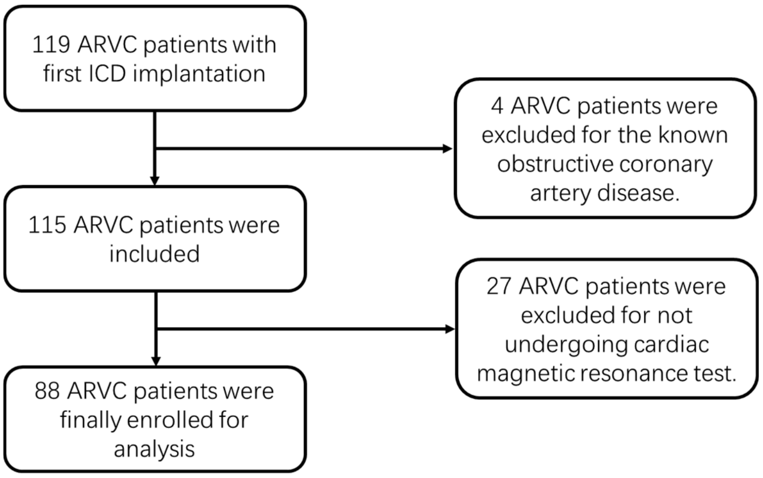

Figure S1. The flowchart of study design. ARVC, arrhythmogenic right ventricular cardiomyopathy; ICD, implantable cardioverter-defibrillator.

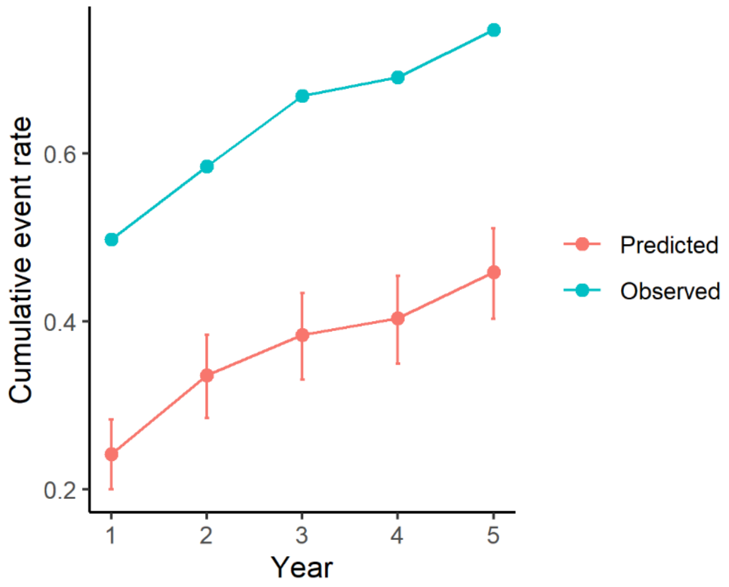

Figure S2. Comparison between model-predicted and observed event rates in total patients.

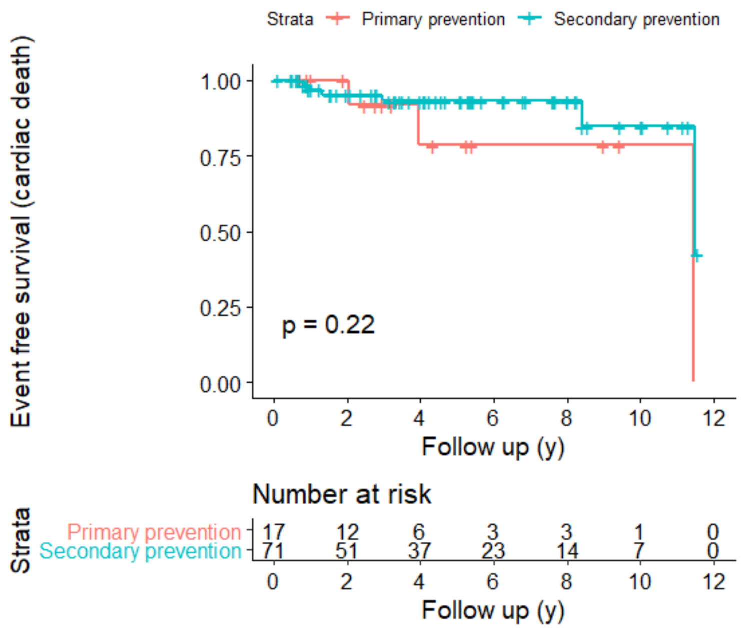

Figure S3. Comparison of the cardiac death stratified by primary prevention and secondary prevention.
